# Synergistic Effects of Biochar and Irrigation on Sugar Beet Growth, Yield, Quality, and Economic Benefit in Arid Regions

**DOI:** 10.3390/plants14030368

**Published:** 2025-01-25

**Authors:** Fuchang Jiang, Yi Li, Liwei Li, Xiangwen Xie, Wanli Xu, Yang Gao, Asim Biswas

**Affiliations:** 1 Key Laboratory of Agricultural Soil and Water Engineering in Arid and Semiarid Areas at Ministry of Education, College of Water Resources and Architectural Engineering, Northwest A&F University, Yangling 712100, China; jiangfuchang@nwafu.edu.cn (F.J.); 18119906527@163.com (L.L.); 2 Institute of Soil Fertilizer and Agricultural Water Saving, Xinjiang Academy of Agricultural Sciences, Urumqi 830091, China; xiexw@sina.cn (X.X.); wlxu2006@163.com (W.X.); 3 Institute of Farmland Irrigation, Chinese Academy of Agricultural Sciences, Xinxiang 453002, China; gaoyang@caas.cn; 4 School of Environmental Sciences, University of Guelph, Guelph, ON N1G 2W1, Canada; biswas@uoguelph.ca

**Keywords:** sugar beet, *SPAD*, germination rate, sustainable yield index

## Abstract

Water scarcity hinders sustainable agriculture in arid and semi-arid regions. This study investigated the combined effects of trickle irrigation and biochar application on sugar beet cultivation in northwest China’s arid and semi-arid regions, addressing challenges of water scarcity. Three-year field experiments were conducted using plastic film mulch, four irrigation levels based on crop evapotranspiration (0.6–1.2 *ETc*), and four biochar application rates (0–30 t ha^−1^). Results showed that biochar application increased sugar beet germination rates by 7.2–24.5% and enhanced relative chlorophyll content by 3.1–22.1%. Optimal combinations of irrigation and biochar significantly improved growth indices and yield, with maximum values observed under the highest irrigation (1.2 *ETc*) and 10 t ha^−1^ biochar treatment. However, the 1.0 *ETc* irrigation treatment with 10 t ha^−1^ biochar demonstrated superior water use efficiency (14.8% higher), sustainable yield index (1.1% higher), and economic benefits (1.4% higher) compared to the highest irrigation treatment. Considering growth, yield, quality, water use efficiency, sustainability, and economic factors, an irrigation level of 1.0 *ETc* combined with a biochar application rate of 10 t ha^−1^ is recommended for sugar beet cultivation in Xinjiang. This study provides valuable insights and practical strategies for water conservation, high yield, and quality improvement in sugar beet cultivation under arid and semi-arid conditions, contributing to sustainable agricultural practices in water-scarce regions.

## 1. Introduction

Sugar beet holds a crucial position in agricultural production as a primary sugar and cash crop, accounting for approximately 30% of the world’s sugar production [1]. Beyond its role in sugar production, molasses derived from sugar beet is extensively utilized across various industries, including chemicals (methanol, ethanol, butanol, and glycerol), pharmaceuticals (vitamins and minerals), and food production (MSG and citric acid) [2]. The cultivation of sugar beet is particularly significant in arid and semi-arid regions where water resources are limited and soil salinity is prevalent. The shortage of water and the issue of soil salinization pose significant challenges to sustainable agricultural development in these areas. Thus, enhancing soil quality and developing effective irrigation strategies for sugar beet cultivation are essential for improving both yield and quality. By addressing these challenges, sustainable sugar beet production can be ensured, contributing to the broader agricultural sustainability in water-scarce regions.

Biochar is a solid material rich in carbon that is produced from organic matter at temperatures ranging from 300 to 1000 °C in the absence of oxygen [3]. As a novel soil amendment, biochar has garnered significant attention for its potential to enhance the soil environment, primarily due to the following reasons: (1) With high specific surface area and porosity, the application of biochar can reduce soil bulk density and increased porosity [4]. (2) Abundant carbon could increase soil activity and soil organic matter content [5]. (3) With good adsorption capacity, it can improve soil cation exchange rate and the utilization efficiency of nutrients and reduce nutrient loss [6]. (4) It can improve the structure of soil aggregates and increase the types and activities of enzymes in soil [7]. (5) It can repair polluted soil while regulating soil acidity and alkalinity [8]. Additionally, biochar also has strong water retention properties, which can inhibit soil water evaporation [9], thus achieving the effect of enhancing soil water content [10].

Biochar not only enhances soil properties but also holds significant potential for increasing crop productivity. Numerous studies have demonstrated that appropriate biochar application rates can enhance the productivity of various crops, including cotton, wheat, maize, rice, and tomato [11,12,13,14,15]. Wang et al. [16] conducted a three-year study on the application of biochar, which showed that biochar had a significant impact on the growth, development, yield, and quality of cotton. However, the application of biochar exceeding 30 t ha^−1^ was not conducive to cotton growth; Liu et al. [17] confirmed in their experiment of intercropping fava bean and ryegrass that the application of biochar can increase leaf moisture content and total aboveground biomass in the intercropping system. Secondly, studies have shown that different types of biochar can significantly affect the application efficiency. Ren et al. [18] found that both the type and the application level of biochar can affect the level of soil improvement and nitrogen uptake by plants; the study by Tiong et al. [19] showed that biochar made from coconut coir significantly enhanced the biological nitrification process and significantly increased the chlorophyll content of tomato plants; Shang et al. [20] found that pyrolysis temperature significantly affects the properties of biochar, especially its surface characteristics.

For sugar beet, there are significant differences in the amount of biochar applied under different planting conditions. Wang et al. [21] found that an application rate of biochar at 20 t ha^−1^ had positive effects on the growth and sugar production of sugar beet in Heilongjiang Province. Li et al. [2] (2022) observed that an application rate of 10 t ha^−1^ resulted in the most significant increase in sugar beet yield, while rates between 10 and 25 t ha^−1^ also showed a positive impact. However, application rates exceeding 30–100 t ha^−1^ led to a decrease in sugar beet yield. Lebrun et al. [22] believed that adding 2% biochar to soil was the most beneficial to the growth of sugar beet, doubling the yield compared to no biochar application. Alves et al. [23] found that when 5% biochar was used instead of conventional fertilizer, sugar beet yield decreased by 45% in sandy loam soils of the Brazilian savanna. Therefore, it is necessary to study the effects of biochar on the growth characteristics of sugar beet in arid and semi-arid salinized soil, especially in the Xinjiang region of China, a region of strong agricultural, economic, and cultural significance.

In addition, as a high-water-demand crop, a reasonable irrigation strategy for sugar beets can not only improve yield and quality but also enhance water use efficiency and reduce water resource waste [24]. However, there are significant differences in the water requirements of sugar beets in different regions. For example, in Central Europe, the annual water demand for sugar beet is about 200 mm. In some Mediterranean countries, the annual water demand for sugar beet is 300–500 mm, while in Asia, North Africa, and South America, the annual water demand for sugar beet is about 600 mm [25]. Specifically, sugar beet yield can only be guaranteed under fully irrigated conditions (approximately 700 mm) in the western United States [26]. In the southern Xinjiang region of China, Yan et al. [27] believed that the highest sugar beet yield was achieved under 0.6 ETc (about 200 mm) irrigation. These studies show that the amount of water required by sugar beets varies greatly from region to region. However, as an important region for sugar beet production in China, there are relatively few studies on the water demand of sugar beet. This study hypothesized that the synergistic effect of biochar and irrigation can effectively promote the growth of sugar beet and enhance yield and quality, resulting in a significant economic effect of sugar beet cultivation. Therefore, the objectives of this study were (1) to explore the effects of different amounts of biochar application and irrigation on the growth, physiological indexes, yield, and quality of sugar beet; (2) to determine the effects of irrigation and biochar application on the stability of sugar beet harvest; and (3) based on economic benefits, to determine the amount of biochar application and irrigation suitable for sugar beet cultivation in Xinjiang.

## 2. Materials and Methods

### 2.1. Experimental Site

The study area was situated in 8th Division, 146th Regiment, 13th company, Shihezi, Xinjiang, China (85°97′05” E; 44°47′83” N). The region experiences an arid climate year-round, typical of the temperate continental climate prevalent in this area. The long-term average temperature is 6.2 °C, with a frost-free period lasting between 168 and 171 days, and the precipitation ranges from 180 to 270 mm. The soil tape is sandy loam (clay, silt, and sand accounted for 3.1%, 69.1%, and 27.8%, respectively), with an average density of 1.53 g cm^−3^ at 0–100 cm soil depth. The soil’s pH is 8.3; the soil’s organic carbon is 8.5 g kg^−1^, and the total nitrogen is 0.4 g kg^−1^. Groundwater is located at >3 m below the surface. Field meteorological data were collected using a portable automatic meteorological station (HOBO U30, MA, USA) which recorded parameters such as rainfall, mean wind speed, average temperature, solar radiation intensity, sunshine duration, and relative humidity levels. During the sugar beet growth periods in 2021, 2022, and 2023, respectively, total precipitations were 91.7 mm, 81.8 mm, and 69.8 mm with corresponding mean temperatures of 20.13 °C, 21.05 °C, and 19.63 °C.

### 2.2. Experimental Design

#### 2.2.1. Biochar Preparation and Experimental Plot Arrangement

The raw material for biochar was derived from the empty shells of palm fruit branches. Biochar was made as follows: to the palm fruit shells in the collection, a blower was used for debris separation measures, followed by the use of dryers to dry the water in the shells that were crushed, and then the raw materials were processed into the carbonization furnace in a closed oxygen-deficient environment for heating to 600 °C for the pyrolysis reaction; for the biocarbon in the carbonization furnace to complete the pyrolysis reaction, it needs to be cooled for more than 8 h so that the temperature is reduced to room temperature. This process was carried out by Zhengzhou Yongbang New Energy Equipment Technology Co. Ltd. (Zhengzhou, China). The diameter of the biochar particles measured approximately 2 mm, effectively enhancing the contact area between the biochar and soil. To mitigate any potential impact on salinized soil pH levels, ferrous sulfate was used to acidify the biochar to a pH value of 6.7. The bulk density was 0.5 g cm^−3^; the specific surface area was 217 m^2^ g^−1^; the water drop penetration time was 8 s, and the cation exchange capacity was 12.2 cmol ka^−1^; the initial electrical conductivity (*EC_1:5_*) was 11.02 mS cm^−1^; the dissolved organic carbon was 143.5 mg kg^−1^; nitrate nitrogen was 2.54 mg kg^−1^; ammonia nitrogen was 1.15 mg kg^−1^; the available potassium was 2575 mg kg^−1^; the total nitrogen was 2.30 g kg^−1^, and the total organic carbon was 472.2 g kg^−1^.

The experiment was conducted in a completely randomized two-factor arrangement consisting of four irrigation rates and four biochar application rates as treatments, with each repeated three times. There were a total of 16 treatments and 48 plots. The dimensions of each test plot were 8 m× 6 m, with a 0.5 m aisle between plots to minimize interferences between the treatments. Biochar application rates of 0, 10, 20, and 30 t ha^−1^ were applied once before sowing in the year 2021, and samples were monitored for three consecutive years.

**Figure 1:**
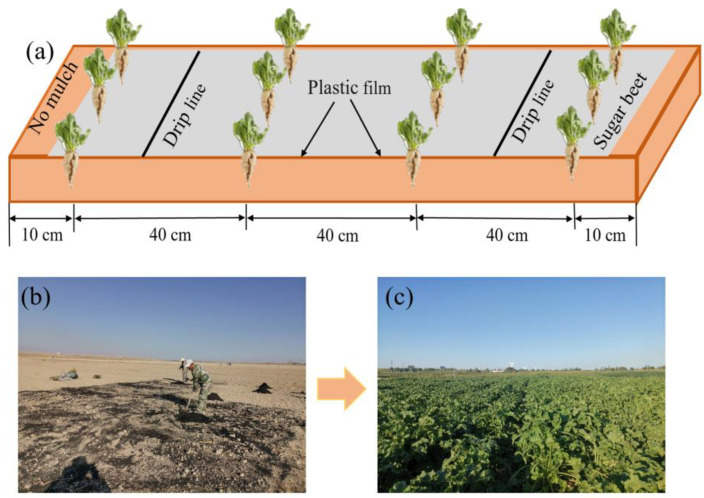
Schematic diagram (**a**) and experimental planting (**b**,**c**) of sugar beet planting under plastic mulched-drip irrigation.

The sugar beet variety was “Detian 2”. The planting and harvest times were 19 April and 28 September, respectively, in 2021; 15 April and 25 September, respectively, in 2022; and 18 April and 23 September, respectively, in 2023. The planting pattern was “one layer of plastic film, two droppers, four rows”, which is a common practice of local farmers. The row spacing of the sugar beet and the spacing between individual plants were 40 cm (Figure 1).

#### 2.2.2. Irrigation Schedule

The time and amount of irrigation varied annually based on local climatic conditions. In 2021, 2022, and 2023, there were 10, 8, and 9 irrigation events, respectively. The first irrigation was applied five days after sowing with an initial amount of 50 mm per event—a practice referred to as “dry sowing wet seedling.” Detailed descriptions of specific irrigation schemes are provided in Table 1.

The irrigation quota of this experiment was determined based on the actual crop evapotranspiration (*ETc*). Irrigation gradients were set as fractions I1 (0.6 *ETc*), I2 (0.8 *ETc*), I3 (1.0 *ETc*), and I4 (1.2 *ETc*) and calculated using the formula developed by Allen et al. [27]: $ETc=Kc\times {ET}_{0}$, where the crop coefficient (*Kc*) of sugar beet varied across different growth stages, while the daily reference crop evapotranspiration (*ET*_0_) was computed using the standard formula [28]:

(1) $${ET}_{0}=\frac{0.408∆\left(R_{n}-G\right)+\gamma \frac{900}{T+273}\mu_{2}\left(e_{s}-e_{a}\right)}{∆+\gamma \left(1+0.34\mu_{2}\right)}$$

where *R_n_* is the net radiation amount (MJ·m^−2^·d^−1^); *G* is soil heat flux density, and it is set to 0 at the daily scale (MJ·m^−2^·d^−1^); *γ* is the humidity meter constant (kPa·C^−1^); *T* is the daily average temperature (℃); *μ*_2_ is the wind speed at 2 m above the ground (m·s^−1^); *e_s_* and *e_a_* are the saturated and actual water vapor pressures (kPa); and Δ is the slope of the saturation vapor pressure curve at air temperature (kPa C^−1^).

Fertilization was integrated into the irrigation processes representing the types and quantities of fertilizers commonly used locally. Specifically, nitrogen (450 kg ha^−1^), phosphorus (265 kg ha^−1^), and potassium (100 kg ha^−1^) were applied using urea (with a minimum nitrogen content of 46%), diammonium phosphate (with at least 46% P_2_O_5_), and potassium sulfate (containing no less than 52% K_2_O). Weeding and pest control of sugar beets during the reproductive period were consistent with the management of local farmers. Due to the mulch cover in the early stage, no weeding operation was required during the seedling stage, the rapid growth stage of sugar beets, and in the late stage of sugar beet growth; manual weeding was required to clear the weeds in the sugar beets as the coverage of sugar beets reached more than 90%. Diseases and pests, such as flea beetles and their disease, of sugar beet mainly appeared in July and August when the weather was hot, and the method of control was to use a sprayer to spray 1200 times diluted bifenthiacloprid and 24% jinggangmycin every 15 days, with a continuously spray pattern for 2–3 times to control the diseases and pests.

### 2.3. Sampling and Measurement of Sugar Beet

#### 2.3.1. Growth Index of Sugar Beet

On the 25th day after seeding, the germination rate (*GR)* of sugar beet was calculated using the following formula [29]:

(2) $$GR=\frac{T_{SE}}{T_{SN}}\times 100$$

where *GR* is germination rate, *T_SE_* is total number of seedlings emerging, and *T_SN_* is total seeding number.

Five sugar beet plants were randomly selected from each plot, and their height and root lengths were measured using a steel ruler with an accuracy of 1 mm. The tuber thickness of sugar beet root blocks was measured using a vernier caliper with an accuracy of 0.01 mm. The measurement dates were the 42nd, 71st, 96th, 118th, and 142nd days after sowing (*DAS*) in 2021; the 41st, 60th, 90th, 120th, and 154th DAS in 2022; and the 41st, 58th, 86th, 111th, and 131st DAS in 2023. All the leaves of the selected plant were measured to calculate the leaf area index (*LAI*). *LAI* was calculated using the following formula [30]:

(3) $$LAI=\frac{N_{P}\times \sum_{i=1}^{m}(\sum_{j=1}^{n}L_{ij}\times W_{ij}\times 0.84)}{m\times 10^{4}}$$

where *N_P_* is the number of sugar beet planting density (plant m^−2^), m is total number of measured plants, n is total leaf number for single plant, *L_ij_* and *W_ij_* are the leaf length and width (cm) on the *ith* plant, *i* and *j* are the *jth* leaf on the *ith* plant, and 0.84 is the conversion coefficient.

The relative chlorophyll content (presented as *SPAD* value) of sugar beet leaves was measured with a handheld chlorophyll instrument (model SPAD-502, Konica Minolta, Tokyo, Japan), and the measurement time was consistent with *LAI*. A portable photosynthetic instrument li-6400xt (Li COR, Lincoln, NE, USA) was used to monitor the photosynthetic capacity of sugar beet leaves. The measurement position was the middle part of sugar beet leaves, and 6 leaves were measured in each treatment.

#### 2.3.2. Yield, Biomass, and Sugar Content

During the sugar beet harvest period, three 6 m^2^ (2 m × 3 m) sample areas were selected in each plot. All sugar beets in the sample area were excavated and weighed to measure the yields. The aboveground and underground parts of sugar beet were dried to a constant weight at 75 °C, and the biomass was weighed and recorded. During the sugar beet harvest period, a Japan Atago PAL-1 (Tokyo, Japan) portable digital display sugar meter was used to measure the sugar content. Sugar beet juice was extracted and placed into the instrument for sugar content analysis. The sugar yield was further calculated as [27]

(4) $$Sugar\;yield\left(t\;{ha}^{-1}\right)=taproot\;yield\left(t\;{ha}^{-1}\right)\times sugar\;content\left(\%\right)$$

#### 2.3.3. Irrigation Water Use Efficiency Indices

Taproot yield irrigation water use efficiency (${IWUE}_{T}$) and sugar yield irrigation water uses efficiency (${IWUE}_{S}$) were calculated as follows [31]:

(5) $${IWUE}_{T}=\frac{Taproot\;yield\;(kg\;{ha}^{-1})}{Irrigation\;amount\;({kg\;ha}^{-1})}$$

(6) $${IWUE}_{S}=\frac{Sugar\;yield\;(kg\;{ha}^{-1})}{Irrigation\;amount\;({kg\;ha}^{-1})}$$

#### 2.3.4. Yield Sustainability

The sustainable yield index (*SYI*), the sustainable biomass index (*SBI*) and the coefficient of variance (*CV*) of the sugar beet yields were used to assess the stability and sustainability of grain yields and biomass. The *SYI* and *SBI* values were calculated as [32]

(7) $$Sustainable\;yieldt\;index\;(SYI)=\frac{\bar{Y}-\sigma }{Y_{max}}$$

(8) $$Sustainable\;biomass\;index\;\left(SBI\right)=\frac{B-\sigma }{B_{max}}$$

(9) $$Coefficient\;of\;variance\left(CV\right)=\left(\sigma /\bar{Y}\right)\times 100$$

where $\bar{Y}$ is the average sugar beet yield (t ha^−1^), σ represents the standard deviations, B is the average biomass (t ha^−1^), and $Y_{max}$ and $B_{max}$ are the maximum sugar beet yield and biomass, respectively.

#### 2.3.5. Economic Benefit Analysis

Net return was calculated as follows [33]:

(10) $$Net\;return=G_{b}-I_{w}-B_{w}-F_{w}-O$$

where $G_{b}$ is the sugar beet yield benefit (CNY ha^−1^), $I_{w}$ is the irrigation water cost (CNY ha^−1^), $B_{w}$ is the cost of biochar (CNY ha^−1^), $F_{w}$ is the fertilizer input (CNY ha^−1^), and O represents the other inputs (CNY ha^−1^). The price of sugar beet was 505, 520, and 530 (CNY t^−1^) in 2021, 2022, and 2023, respectively. The prices of irrigation water, biochar, and fertilizer were 0.3 CNY m^−3^, 2100 CNY ha^−1^, and 2140 CNY ha^−1^, respectively. The cost of the other inputs including machinery, labor, seeds, drip tapes, plastic mulch, and material was about 11,100 CNY ha^−1^.

### 2.4. Statistical Analysis

Microsoft Excel 2019 (Microsoft Corporation, NM, USA) was used to summarize and analyze the test data. The SPSS 26.0 statistical software (IBM, SPSS, Statistics 26.0) was used to analyze a two-factor (Irrigation amount and biochar application) analysis of variance (*ANOVA*) and Duncan multiple range test (*p* < 0.05 or *p* < 0.01). Utilizing the R package (corrplot) for obtaining correlation coefficients and analyzing the interrelationships among indicators, the entropy–TOPSIS multi-objective decision analysis method was employed to ascertain the weightings of indices and comprehensive evaluation levels for each treatment. Figures were drawn using the Origin 9.0 software.

## 3. Results

### 3.1. Effects of Biochar and Irrigation on the Germination Rate and Growth of Sugar Beet

#### 3.1.1. Influence of Biochar Application Amount on Germination Rate

**Figure 2:**
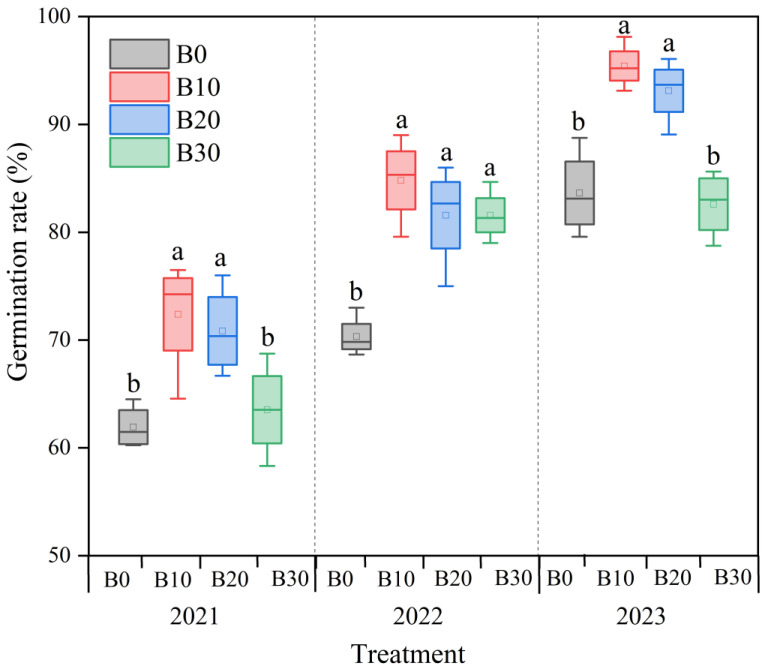
Effect of biochar application on the germination rate of sugar beet. Different letters above the bars indicate statistical differences among treatments at the significance level *p* < 0.05 with an LSD test.

Under consistent irrigation amounts, the one-time biochar application in 2021 led to a gradual increase in the sugar beet germination rate from 2021 to 2023 (Figure 2), ranging 60.3–75.0%, 68.3–84.7%, and 79.6–95.4%, respectively. The highest germination rate was consistently observed under the B10 treatment. Compared to the control (B0), the germination rates for the B10, B20, and B30 treatment groups increased by 19.9–24.5%, 14.1–21.4%, and 7.2–18.9% over the three years, respectively.

#### 3.1.2. Synergistic Effects of Biochar and Irrigation on Growth of Sugar Beet

**Figure 3:**
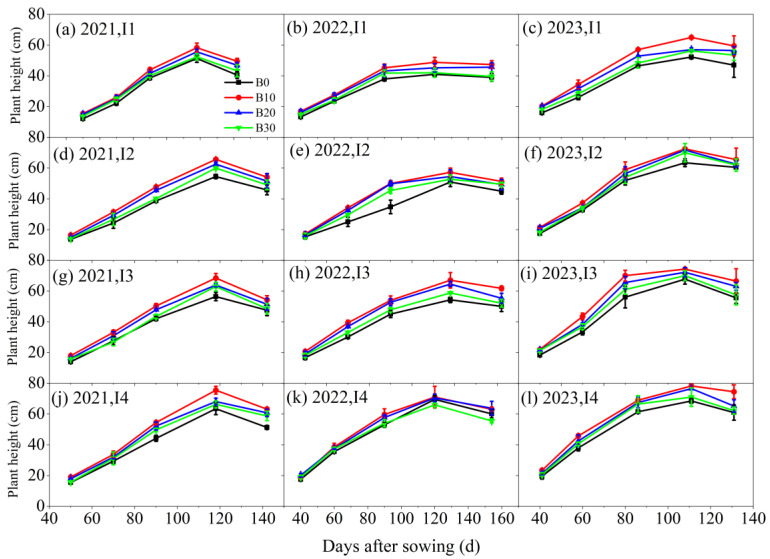
Plant height of sugar beet under different irrigation amounts and biochar application rates in 2021, 2022, and 2023.

The changes in sugar beet plant height under varying irrigation levels and biochar application rates are presented in Figure 3. Throughout the three-year experiment, sugar beet plant height initially increased with the number of sowing days, peaking before eventually declining. The highest plant height was consistently observed under the I4B10 treatment. Biochar application significantly contributed to increased plant height under consistent irrigation conditions. In 2021, the plant heights for the B10, B20, and B30 treatments increased by 20.6%, 13.8%, and 6.9%, respectively, compared to the B0 treatment. Similarly, under the same biochar application rates, plant height increased with higher irrigation levels. In 2021, plant heights under the I2, I3, and I4 irrigation treatments increased by 13.1%, 17.3%, and 26.7%, respectively, compared to the I1 treatment. This trend was consistently observed in 2022 and 2023, demonstrating the beneficial effects of both biochar application and increased irrigation on sugar beet plant height.

**Figure 4:**
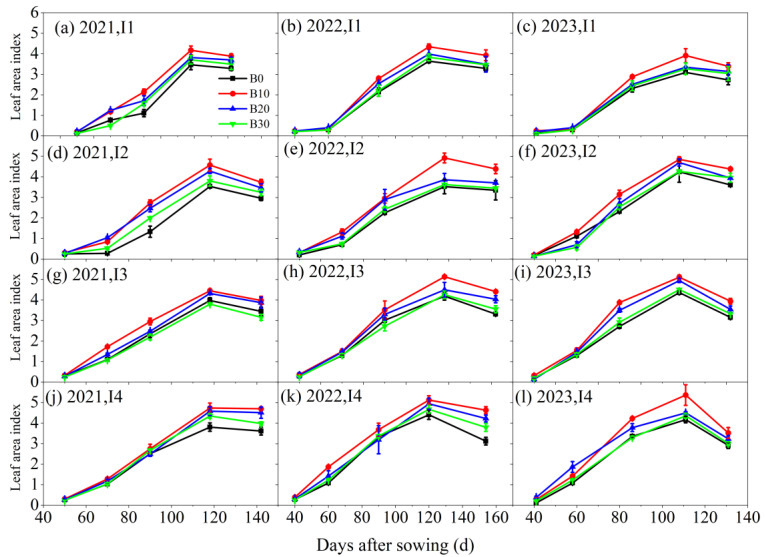
Leaf area index of sugar beet under different irrigation amounts and biochar application rates in 2021, 2022, and 2023.

The *LAI* of sugar beet was influenced by both irrigation amounts and biochar application rates (Figure 4). Over the three-year experiment, *LAI* increased with higher irrigation levels and exhibited an initial rise followed by a decline with increasing biochar application rates, peaking under the I4B10 treatment. Under consistent irrigation conditions, *LAI* in the B10, B20, and B30 biochar treatments increased by 32.6–42.1%, 20.2–35.1%, and 2.8–8.7%, respectively, compared to the B0 treatment without biochar. Similarly, at a constant biochar application rate, *LAI* under the I2, I3, and I4 irrigation treatments increased by −6.8–29.5%, 0.7–15.3%, and 3.2–16.8%, respectively, compared to the I1 treatment. These data highlight the synergistic effects of irrigation and biochar application on improving the *LAI* of sugar beet.

**Figure 5:**
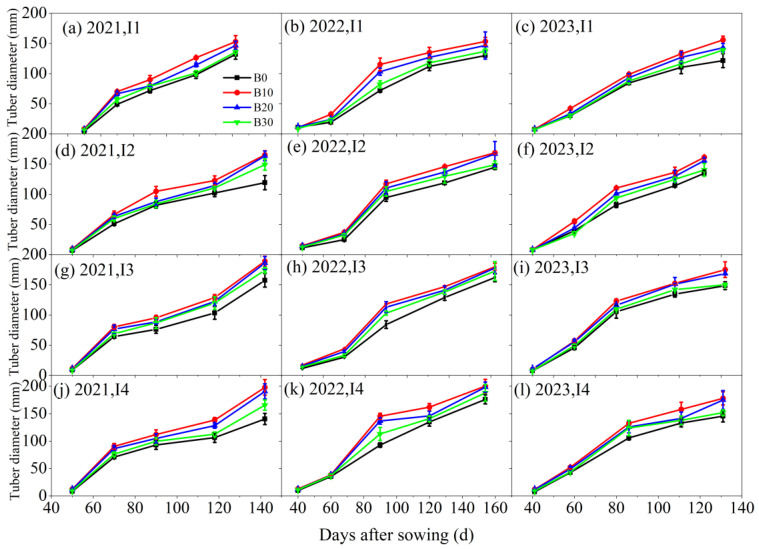
Tuber diameter under different irrigation amounts and biochar application rates in 2021, 2022, and 2023.

**Figure 6:**
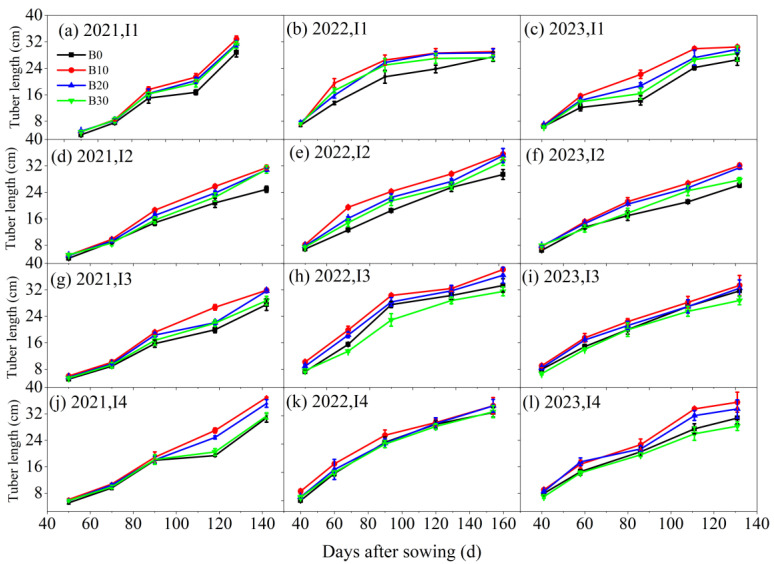
Tuber length of sugar beet under different irrigation amounts and biochar application rates in 2021, 2022, and 2023.

Under varying irrigation levels and biochar application rates, the tuber diameter and tuber length of sugar beet reached their maximum under the I4B10 treatment, both increasing with the number of sowing days (Figure 5 and Figure 6). Biochar application led to a 5.6%, 19.2%, and 6.3% increase in tuber diameter under the I2, I3, and I4 irrigation treatments, respectively, compared to the I1 treatment. Similarly, with the same irrigation amount, the B10, B20, and B30 biochar treatments resulted in a 31.2%, 22.4%, and 12.3% increase in tuber diameter, respectively, compared to the B0 treatment. Additionally, under the same irrigation amount, root length followed the trend B10 > B20 > B30 > B0. Conversely, with the same biochar application rate, tuber length increased with irrigation amounts in the order I4 > I3 > I2 > I1.

### 3.2. Effects of Biochar and Irrigation on Physiological Indexes

#### Effects of Irrigation and Biochar Application on *SPAD*

Table 2 presents the effects of irrigation and biochar application on the *SPAD* values of sugar beet during the seedling stage, the rapid leaf growth stage, the sugar accumulation stage, and the harvest stage in 2021, 2022, and 2023. Throughout the growth stages, *SPAD* values generally increased initially and then decreased, peaking during the sugar accumulation period. Under consistent irrigation conditions, increasing biochar application significantly enhanced *SPAD* values. In 2021, *SPAD* values for the B10, B20, and B30 treatments increased by 8.1–13.5%, 11.5–14.8%, and 13.1–22.1%, respectively, compared to the B0 treatment. In 2022 and 2023, SPAD values increased by 6.2–9.3%, 6.8–13.4%, and 11.9–14.1%, and by 3.1–7.7%, 10.0–13.6%, and 11.3–17.8%, respectively. With the same biochar application rates, *SPAD* values under the I2, I3, and I4 irrigation treatments increased by 1.6% and 0.3% and decreased by 0.3% compared to the I1 treatment, respectively. Statistical analysis over the three-year period indicated that the irrigation amount significantly affected *SPAD* values only during the rapid leaf growth and sugar accumulation stages, while biochar application had a significant impact on *SPAD* values throughout all growth stages. The interaction between irrigation and biochar (I×B) showed significant differences in *SPAD* values only during the rapid leaf growth period.

**Figure 7:**
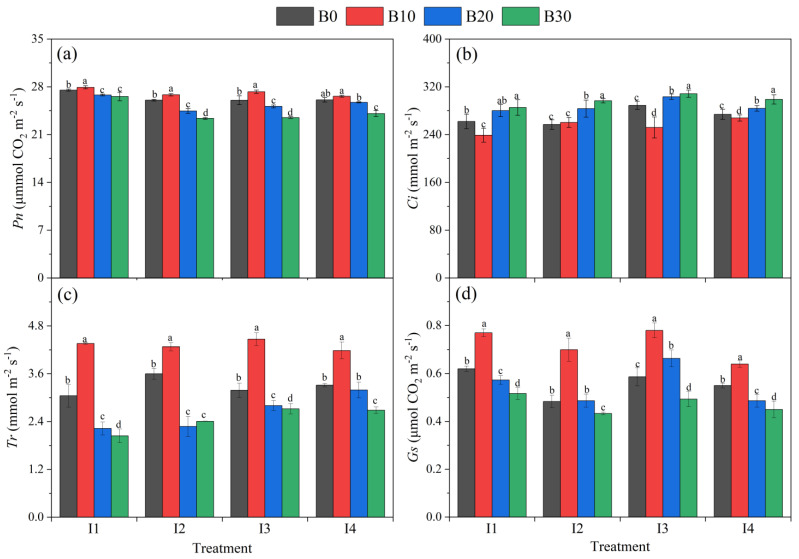
(**a**–**d**) Net photosynthetic rate (*Pn*), stomatal conductance (*Gs*), intercellular carbon dioxide (*Ci*), and transpiration rate (*Tr*) of sugar beet under different irrigation amounts and biochar application rates. Error bars represent standard errors. Different letters above the bars indicate statistical differences among treatments at the significance level *p* < 0.05 with an LSD test.

Figure 7 shows the effects of different irrigation amounts and biochar application rates on net photosynthetic rate (*Pn*), stomatal conductance (*Gs*), intercellular carbon dioxide concentration (*Ci*), and transpiration rate (*Tr*) of sugar beet. With the same amount of irrigation, *Pn* first increased and then decreased with increasing biochar application rates, reaching its maximum value under the B10 treatment (Figure 7a). In contrast, *Ci* exhibited an opposite trend, increasing with higher biochar application rates. Under the B10, B20, and B30 treatments, *Ci* increased by an average of −5.6%, 6.5%, and 10.1% compared to the B0 treatment (Figure 7b). Under consistent irrigation conditions, both *Tr* and *Gs* achieved their highest values under the B10 treatment, increasing by 18.7–42.9% and 16.4–44.8%, respectively, compared to the B0 treatment. *Tr* decreased by 3.7–36.8% and 14.6–33.1% in the B20 and B30 treatments, respectively, compared to the B0 treatment, while *Gs* decreased by 0.7–13.1% and 10.4–18.2%, respectively (Figure 7c,d). With the same biochar application rate, Tr and *Gs* initially increased and then decreased with higher irrigation amounts, reaching their maximum values under the I3 irrigation level.

### 3.3. Effects of Irrigation and Biochar Application Yield, Biomass, Quality, and IWUE

The irrigation amount and biochar application significantly impacted the yield and biomass of sugar beet during the harvesting period, although the interaction between irrigation amount (I) and biochar application amount (B) did not significantly affect the dry matter content (Table 3). Across all treatments, the highest sugar beet production was observed at I4B10, with yields of 130.7, 147.6, and 141.4 t ha^−1^ in 2021, 2022, and 2023, respectively. Under consistent biochar application rates, increased irrigation water enhanced sugar beet yield. Compared to the I1, I2, and I3 treatments, the yield under the I4 treatment increased by an average of 68.4%, 39.9%, and 12.8%. Interestingly, both *IWUE_T_* and *IWUE_S_* decreased with higher irrigation amounts. Compared to I1, I2, and I3, *IWUE_T_* under the I4 treatment decreased by 2.8%, 0.6%, and 2.7%, while *IWUE_S_* decreased by 19.4%, 10.5%, and 10.7%. The use of biochar also increased sugar beet yield; however, when the biochar application rate exceeded 20 t ha^−1^, there was a decrease in yield with further increases in biochar application. Conversely, this trend was observed for *IWUE_T_* and *IWUE_S_* (Table 4).

### 3.4. Variation in Sugar Content

**Figure 8:**
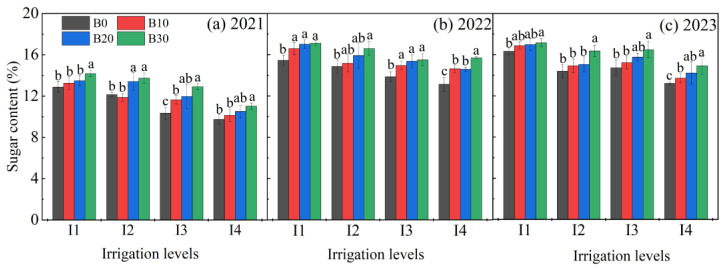
Sugar content under different irrigation amounts and biochar application rates in 2021, 2022, and 2023. Error bars represent standard errors. Different letters above the bars indicate statistical differences among treatments at the significance level *p* < 0.05 with an LSD test.

Both irrigation levels and biochar application significantly influenced the sugar content in sugar beet (Figure 8). Under the same irrigation level, sugar content increased with higher biochar application rates, with the B30 treatment yielding significantly higher sugar content than the B0 (no biochar) treatment. In 2021, the sugar content of sugar beet under the B10, B20, and B30 treatments increased by 4.3%, 9.7%, and 15.3%, respectively, compared to the B0 treatment. In 2022, these treatments resulted in increases of 9.0%, 11.7%, and 15.4%, and in 2023, by 3.7%, 5.7%, and 10.9%, respectively. Conversely, under the same biochar application rate, sugar content decreased with increasing irrigation amounts over the three years. In 2021, sugar content under the I1 irrigation level increased by an average of 4.9%, 12.9%, and 22.8% compared to the I2, I3, and I4 treatments, respectively. This trend continued in 2022 and 2023, with increases of 7.1%, 9.8%, and 12.4%, and 9.8%, 7.6%, and 16.7%, respectively.

### 3.5. Effects of Irrigation and Biochar Application on SYI

The variations in the *SYI*, *SBI*, and the *CV* of sugar beet yield and biomass under different irrigation amounts and biochar application rates are presented in Table 5. Under consistent irrigation conditions, both SYI and SBI initially increased and then decreased with increasing biochar application rates, reaching their highest values under the B10 treatment. Conversely, *CV* for yield (*CVy*) and biomass (*CVb*) first decreased and then increased, hitting their lowest points under the B10 treatment. With a fixed biochar application rate, *SYI* and *SBI* achieved their maximum values under the I3 irrigation treatment, while *CV* reached its minimum under the same treatment. Compared to the I1, I2, and I4 treatments, the I3 irrigation treatment increased SYI and SBI by 1.1–11.6% and decreased *CV* by 0.3–8.2%.

### 3.6. Economic Benefit (EB) Analysis

**Figure 9:**
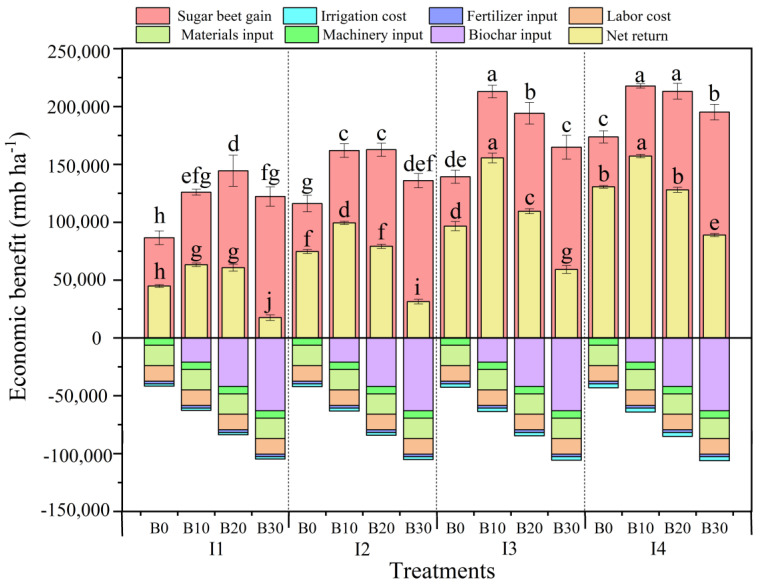
Economic benefit of sugar beet cotton under different irrigation amounts and biochar application rates in 2021, 2022, and 2023. Error bars represent standard errors. Different letters above the bars indicate statistical differences among treatments at the significance level *p* < 0.05 with an LSD test.

Figure 9 shows the cost, revenue, and net profit of sugar beet production from 2021 to 2023 under different treatments. Under the same irrigation levels, *EB* initially increased and then decreased with higher biochar application. The highest values under both the I1 and I2 irrigation amounts were reached under the B20 treatment; however, the maximum EB was reached with the B10 treatment under the I3 and I4 irrigation levels. Over the three years, the *EB* for the B10, B20, and B30 biochar treatments increased by 38.8%, 13.2%, and −47.4%, respectively, compared to the B0 treatment. Under the same biochar application rate, increasing irrigation amounts enhanced the *EB* of sugar beet and reached its maximum at the I4 irrigation level.

### 3.7. Comprehensive Evaluation

**Figure 10:**
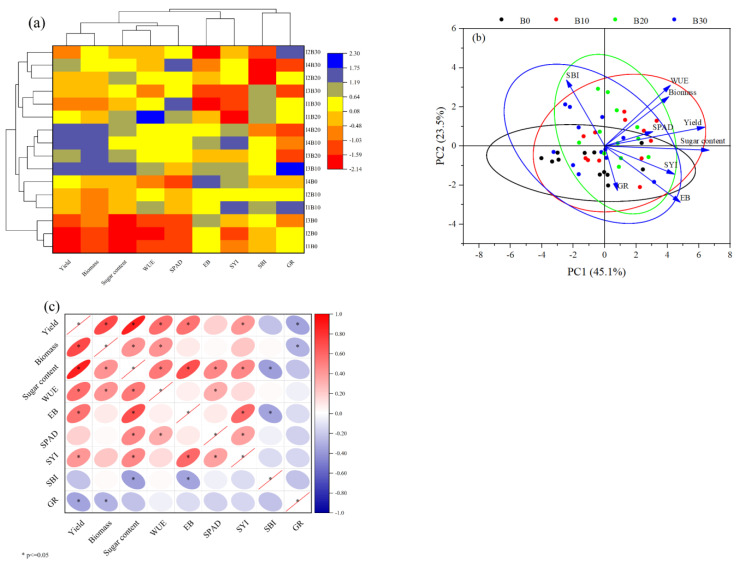
Heat maps (**a**), principal component analysis (**b**), and correlation analysis (**c**) for different relevant indicators under different irrigation and biochar treatments in 2021, 2022, and 2023. *WUE*, irrigation water use efficiency; *EB*, economic benefit; *SYI*, sustainable yield index; *SBI*, sustainable biomass index; *GR*, germination rate; *, *p* <0.05.

Figure 10 shows the relationships among various indices, such as beet emergence rate, yield, *WUE*, *EB*, and *SYI*, under different combinations of irrigation and biochar treatments. The data indicate that the I3B10 treatment consistently resulted in higher levels of yield, biomass, and sugar content. Treatments I3B10, I3B20, I4B10, and I4B20 clustered together, with I3B10 exhibiting superior *WUE*, *EB*, and *SYI*. Conversely, treatments I1B0, I2B0, and I3B0 formed a distinct group, underscoring the catalytic effects of biochar on sugar beet growth (Figure 10a). Principal component analysis (*PCA*) revealed that yield, biomass, *WUE*, *SPAD*, sugar content, and *SYI* were closely interrelated. Although *GR* can enhance sugar beet yield, its effect was not significant (Figure 10b). A comprehensive analysis of various indices identified the B10 biochar application as the most favorable treatment (Figure 10c).

## 4. Discussion

### 4.1. Effects of Biochar Application on Germination Rate

Germination rate is a critical factor influencing crop yield, as it is affected by various factors [34,35]. Understanding the impact of biochar application on crop germination rate is essential for promoting crop growth, increasing yield, and achieving higher economic benefits. Bai et al. [36] demonstrated that biochar produced through pyrolysis at 200 °C had a more pronounced stimulatory effect on seed germination compared to biochar produced at 500 °C and 800 °C. Additionally, they found that higher application rates of biochar significantly decreased crop germination rates. Conversely, Wang et al. [37] discovered that biochar produced from almond shells at 550 °C was more conducive to cucumber germination, with a significantly higher germination rate at 60 t ha^−1^ compared to 30 t ha^−1^.

Das et al. [38] studied the impact of biochar on maize and rye germination rates, revealing significant increases at an application rate of 10 t ha^−1^ compared to 5 t ha^−1^ and no biochar treatment. Similarly, Bouqbis et al. [39] reported that biochar application significantly enhanced barley germination rates and increased the fresh weight of barley seedlings. Interestingly, Quilliam et al. [40] found that a single application of biochar did not significantly affect weed germination rates, but continuous biochar application significantly reduced weed germination rates.

Li et al. [2] observed that after three years of continuous biochar application, the germination rate of sugar beet significantly improved at an application rate of 10 t ha^−1^ but decreased with higher application rates. However, continuous biochar application did not lead to a significant improvement in the germination rate of sugar beet. Our study corroborates these findings, as shown in Figure 2. The results indicate that biochar application can enhance the germination rate of sugar beet, with the optimal application amount being 10 t ha^−1^. Additionally, we found that the germination rate of sugar beet increased with prolonged biochar application duration, underscoring the potential benefits of biochar in sustainable agriculture practices.

### 4.2. Synergistic Effects of Biochar and Irrigation on Chlorophyll

Chlorophyll serves as a crucial indicator of nutrient absorption capacity and crop growth status [41]. Its production is influenced by various factors, including irrigation, fertilizers, and environmental conditions [42]. The role of irrigation in plant growth has been extensively studied for its impact on chlorophyll levels [43,44]. Bekhradi et al. [45] found no significant difference in basic chlorophyll content with varying irrigation amounts, but under deficit irrigation conditions (50% of field capacity), chlorophyll content showed a decreasing trend. Wei et al. [46] reported that nitrogen levels and crop growth periods were determinants of chlorophyll content, whereas irrigation levels did not significantly affect it. Conversely, Dos et al. [47] discovered that chlorophyll content significantly increased with higher irrigation levels under conditions ranging from 25% to 100% *ETc*. Similarly, Tourajzadeh et al. [48] demonstrated that chlorophyll content in quinoa was significantly higher under 100% crop water irrigation compared to 60% irrigation, with no significant difference observed compared to 80% irrigation.

Dos et al. [49] also found that increasing both irrigation and nitrogen application elevated chlorophyll content and photosynthetic rates in *Tithonia diversfolia*. This trend was similarly observed by Li et al. [50] throughout the growth period of winter wheat. Our study corroborates these findings, showing that chlorophyll content increases with higher irrigation levels (60–100% *ETc*) but reaches its lowest point at 120% irrigation level (Table 3). Excessive irrigation led to overgrowth of branches and leaves, likely due to insufficient nutrient supply, consistent with findings by Norouzi et al. [51].

Moreover, our study revealed that chlorophyll content increased with higher biochar application, consistent with the results reported by Li et al. [52]. Biochar application has been shown to significantly increase leaf chlorophyll content. Previous research indicated that biochar is rich in various organic compounds, enhancing soil organic matter and facilitating nutrient uptake in crops [53]. Feng et al. [54] demonstrated a nearly 30% increase in maize chlorophyll content with a biochar application rate of 50 t ha^−1^. Tiong et al. [19] reported a significant increase in tomato chlorophyll content under biochar-treated conditions compared to those without biochar treatment. However, Akhtar et al. [55] observed a significant decrease in tomato chlorophyll content with a biochar application rate of 5 t ha^−1^, consistent with Kammann et al. [56] in their study of wild quinoa. The increase in the soil C/N ratio following biochar application can result in nitrogen fixation, reducing nitrogen absorption by plants, which may be the primary reason for the observed decrease in chlorophyll content [57].

### 4.3. Synergistic Effects of Biochar and Irrigation on Growth and Yield of Sugar Beet

The amount of irrigation and biochar application significantly affected the yield and biomass of sugar beet (Table 3). Appropriate irrigation promotes the development of crop roots, accelerates plant growth, and improves yield and *WUE* [45]. Li et al. [58] demonstrated that increasing irrigation can enhance crop yield, but excessive irrigation significantly reduces WUE. Liu et al. [59] also confirmed that increased irrigation improves water and fertilizer utilization efficiency in wheat, thereby increasing its production. Conversely, water deficits hinder growth and crop development, shorten the growth cycle, and reduce yield and biomass [60].

This experiment’s findings indicated that higher irrigation levels contribute to enhanced sugar beet yield, with yields under 1.2 *ETc* irrigation significantly surpassing those under the 0.6 *ETc* and 0.8 *ETc* irrigation levels, but the difference is not significant compared to the 1.0 *ETc* irrigation level. The results are consistent with Yan et al. [26]. And differences in soil types, climatic conditions, and management practices can influence sugar beet water demand [61].

Biochar application has been shown to enhance plant growth and increase yield [55,62]. The promotional effect of biochar on plant growth is largely contingent on its physical structure and properties. Increased porosity and reduced bulk density effectively enhance gas exchange and nutrient absorption by plant roots [4]. Additionally, biochar augments soil water holding capacity and improves plant water status [63]. It also enhances soil organic matter and other nutrients, facilitating plant absorption and leading to increased yield [13,64]. However, excessive biochar application can be detrimental. Li et al. [2] observed a decline in sugar beet yield when biochar application exceeded 10 t ha^−1^. Similarly, Wang et al. [36] found that cotton yield initially increased with biochar application rates up to 25 t ha^−1^ but decreased beyond this threshold. Feng et al. [65] noted a similar trend in maize, with yields declining when biochar application exceeded 30 t ha^−1^. These studies confirm that while biochar can benefit soil and crop growth, it is essential to apply the appropriate amount. This study also found that the application of biochar significantly increased sugar beet yield and biomass (Table 3), and the application of biochar treatment resulted in an average increase of 15.73% in yield compared to the treatment without biochar addition.

Considering factors such as crop yield, economic benefits, *WUE*, and yield stability index, we recommend an irrigation level of 1.0 *ETc* and a biochar application rate of 10 t ha^−1^ as the optimal combination for arid and semi-arid regions. However, to fully understand the long-term effects of biochar application in Xinjiang and similar ecosystems, factors such as biochar type, raw material availability, and preparation process must be evaluated. Additionally, subsequent long-term experiments should address soil properties, nutrient availability, water management, and fertilizer use efficiency.

The application of biochar increased the seedling rate of sugar beet by 7.2–24.5%. The growth indexes of beet (stem diameter, plant height, leaf area index, and root length) increased with the increase in irrigation amount; it first increased and then decreased with the increase in biochar application amount, and all reached the maximum value under the I4B10 treatment. With the increase in biochar application amount, *SPAD* increased by 3.1–22.1%, but irrigation amount had no significant effect on *SPAD*. Under the combination of biochar and irrigation, the sugar beet yield reached the maximum under the I4B10 treatment, and there was no significant difference in sugar beet yield between I4B10 and I3B10. However, under the I3 irrigation treatment, *WUE*, *SYI*, and *EB* increased by 14.8%, 1.1%, and 1.14% compared with the I4 treatment, respectively. Considering the factors of sugar beet growth, yield, quality, *WUE*, and *EB*, the irrigation level set to 1.0 *ETc* and the application amount of biochar set to 10 t ha^−1^ could be used as the recommended scheme for sugar beet planting in Xinjiang region. In order to comprehensively assess the specific impacts of irrigation and biochar application on crops, it is essential to conduct longer-term observations and consider additional factors such as nutrient availability and fertilizer utilization.

## Figures and Tables

**Table 1:** Irrigation amounts over the three growth seasons of sugar beet in 2021, 2022, and 2023.

Irrigation Times	Days After Sowing (d)	*ET_0_* (mm)	*K_C_*	Irrigation Amount (mm)			
				I1 (0.6 *ETc*)	I2 (0.8 *ETc*)	I3 (1.0 *ETc*)	I4 (1.2 *ETc*)
2021							
1	5	-	-	50	50	50	50
2	20	-	-	25	25	25	25
3	55	36	0.7	16	20	25	30
4	66	37	0.7	16	21	26	31
5	73	51	1.05	32	43	54	64
6	86	41	1.05	26	35	43	52
7	98	41	1.05	26	35	43	52
8	107	41	0.95	23	31	39	47
9	113	23	0.95	13	17	22	26
Total				227	277	327	377
2022							
1	5	-	-	50	50	50	50
2	55	50	0.7	21	28	35	42
3	66	55	0.7	23	31	39	46
4	73	35	1.05	22	29	38	44
5	86	55	1.05	35	46	58	69
6	98	56	1.05	35	47	59	70
7	107	52	0.95	30	40	49	59
8	113	32	0.95	18	24	30	36
Total				234	295	358	416
2023							
1	5	-	-	50	50	50	50
2	44	50	0.7	21	28	35	42
3	54	38	0.7	16	21	27	32
4	66	46	1.05	29	39	48	58
5	79	33	1.05	21	28	35	42
6	91	40	1.05	25	34	42	50
7	103	31	0.95	18	24	30	35
8	110	23	0.95	13	18	22	26
9	117	24	0.95	14	18	23	27
Total				207	260	312	362

**Table 2:** *SPAD* values under different irrigation and biochar combinations in 2021, 2022, and 2023.

Treatment	2021				2022				2023			
	Seedling	Leaf Fast Growth	Root Sugar Increment	Harvesting	Seedling	Leaf Fast Growth	Root Sugar Increment	Harvesting	Seedling	Leaf Fast Growth	Root Sugar Increment	Harvesting
I1B0	34.7 c	38.4 c	44.3 b	41.0 a	37.9 c	53.3 b	53.4 b	46.1 c	37.3 c	54.7 c	50.1 c	45.2 c
I1B10	36.6 b	40.8 b	51.4 a	45.8 c	47.2 a	55.2 a	54.5 b	51.6 b	43.3 a	59.9 b	52.3 b	47.2 b
I1B20	36.8 b	40.6 b	52.4 a	50.9 a	44.7 b	55.2 a	56.4 ab	54.1 a	42.9 a	59.8 b	56.9 a	49.6 ab
I1B30	38.8 a	43.2 a	52.3 a	48.3 b	45.9 ab	56.5 a	57.6 a	53.3 a	41.2 b	60.0 a	57.2 a	51.2 a
I2B0	37.9 c	40.5 c	42.3 b	41.3 c	39.2 b	51.5 c	56.2 b	47.3 b	38.5 c	51.8 d	52.8 c	44.8 c
I2B10	39.1 b	42.1 b	48.8 a	45.3 b	44.4 a	55.5 b	60.8 a	51.4 a	41.8 b	55.6 c	55.3 b	47.1 b
I2B20	40.5 ab	43.2 ab	49.3 a	47.6 a	43.3 a	59.6 a	60.6 a	52.3 a	44.5 a	63.1 b	57.2 ab	48.7 b
I2B30	41.8 a	44.6 a	49.6 a	47.2 a	42.7 ab	60.8 a	61.3 a	52.9 a	44.2 a	68.6 a	58.0 a	50.8 a
I3B0	40.0 b	40.0 c	44.3 c	40.4 c	37.9 b	53.2 c	52.5 c	44.0 c	40.3 c	49.6 d	49.2 b	45.0 c
I3B10	42.1 a	42.9 b	48.2 b	45.0 b	41.2 a	55.8 b	57.6 b	49.6 b	42.4 b	53.5 c	50.6 b	46.5 c
I3B20	42.5 a	44.7 a	48.6 b	48.1 a	42.2 a	58.7 a	58.8 ab	53.0 a	42.3 b	55.6 b	55.9 a	48.8 b
I3B30	43.2 a	45.3 a	52.2 s	49.8 a	42.6 a	59.1 a	59.1 a	53.3 a	46.6 a	59.5 a	56.1 a	53.9 a
I4B0	36.1 c	38.9 c	42.4 c	40.5 c	39.2 b	56.2 b	55.9 c	43.6 c	37.7 c	50.1 b	50.3 b	46.7 c
I4B10	42.8 a	44.4 b	46.6 b	46.3 b	42.1 ab	59.3 ab	58.7 b	47.4 b	40.3 b	52.1 b	50.6 b	47.5 bc
I4B20	41.4 b	45.1 ab	47.3 b	46.9 b	42.2 ab	61.4 a	58.8 b	45.7 b	44.2 a	56.1 a	54.2 a	48.8 b
I4B30	43.1 a	46.8 a	51.2 a	51.6 a	43.4 a	61.8 a	62.6 a	50.7 a	44.8 a	54.8 a	55.4 a	53.0 a
ANOVA												
I	*	**	**	NS	*	**	**	**	NS	**	**	NS
B	**	**	*	**	**	**	**	**	**	**	**	**
I × B	**	**	**	**	NS	*	NS	NS	*	**	NS	NS

Note: Different letters represent statistical differences between treatments with a significance level of *p* < 0.05 in the LSD test; * indicates that it is significant at the level of 0.05; ** indicates that it is significant at the level of 0.01; I is the irrigation amount; B represents the biochar application rates.

**Table 3:** Sugar beet yield, biomass, and sugar yield under different irrigation and biochar combinations in 2021, 2022, and 2023.

Treatment	2021			2022			2023		
	Taproot Yield (t ha^−1^)	Biomass	Sugar Yield	Taproot Yield	Biomass	Sugar Yield (t ha^−1^)	Taproot Yield	Biomass (t ha^−1^)	Sugar Yield (t ha^−1^)
		(t ha^−1^)	(t ha^−1^)	(t ha^−1^)	(t ha^−1^)		(t ha^−1^)		
I1B0	58.5 d	15.7 c	7.5 c	53.7 d	16.2 ab	8.3 d	54.8 b	13.6 a	8.9 c
I1B10	89.5 b	16.9 b	11.8 b	66.2 c	17.6 a	10.9 c	87.4 a	14.2 a	14.8 a
I1B20	108.6 a	20.2 a	14.6 a	90.8 a	17.2 a	15.4 a	79.8 a	13.1 a	13.5 ab
I1B30	79.4 c	16.7 b	11.2 b	80.1 b	16.7 b	13.7 b	76.4 ab	11.3 b	13.1 b
I2B0	61.5 b	13.9 d	7.4 c	85.5 c	17.2 b	11.8 c	76.6 c	14.2 c	11.0 c
I2B10	93.2 a	16.4 c	11.1 b	120.3 a	19.5 a	19.4 a	98.3 a	18.6 a	13.5 b
I2B20	99.9 a	20.2 b	13.4 a	117.2 b	19.9 a	19.6 a	90.8 b	15.7 b	13.7 b
I2B30	86.2 ab	21.1 a	11.8 ab	85.2 c	18.0 b	14.1 b	90.6 b	18.8 a	14.8 a
I3B0	74.5 c	17.1 d	7.7 c	106.4 c	18.1 b	14.7 c	87.4 c	13.4 c	12.8 c
I3B10	128.5 a	26.5 b	14.9 a	147.3 a	19.9 a	23.2 a	140.5 a	24.6 a	20.5 a
I3B20	117.8 a	28.3 a	14.1 a	128.0 b	19.8 a	19.6 b	132.7 b	23.8 a	20.3 a
I3B30	95.4 b	19.7 c	12.3 b	115.5 b	18.9 b	17.9 b	106.7 b	17.7 b	17.1 b
I4B0	112.8 b	18.7 c	11.0 b	115.8 c	18.5 ab	15.2 b	106.3 b	22.7 a	14.1 c
I4B10	130.7 ab	26.6 a	13.2 a	147.6 a	21.5 ab	22.6 a	141.3 a	22.8 a	19.4 a
I4B20	128.5 a	27.6 a	13.5 a	143.6 b	21.8 a	20.9 a	138.7 a	20.5 b	19.7 a
I4B30	119.1 c	22.1 b	13.1 a	140.1 b	19.5 b	21.9 a	117.2 b	19.3 b	17.5 b
ANOVA									
I	**	*	**	**	*	**	**	**	**
B	**	**	**	*	*	**	**	**	**
I×B	*	NS	**	NS	NS	**	**	NS	**

Note: Different letters represent statistical differences between treatments with a significance level of *p* < 0.05 in the LSD test; * indicates that it is significant at the level of 0.05; ** indicates that it is significant at the level of 0.01; I is the irrigation amount; B represents the biochar application rates.

**Table 4:** Irrigation and biochar application treatments on *IWUE_T_* and *IWUE_S_* in 2021, 2022, and 2023.

Treatment	2021		2022		2023	
	*IWUE_T_*	*IWUE_S_*	*IWUE_T_*	*IWUE_S_*	*IWUE_T_*	*IWUE_S_*
I1B0	25.79 d	3.32 c	22.98 d	3.56 d	21.09 c	3.44 c
I1B10	39.43 b	5.22 b	28.29 c	4.70 c	33.63 a	5.72 a
I1B20	47.86 a	6.46 a	38.84 a	6.62 a	30.72 b	5.19 b
I1B30	34.98 c	4.96 b	34.25 b	5.87 b	29.39 b	5.05 b
I2B0	22.26 c	2.71 v	28.99 b	4.02 c	23.57 b	3.39 b
I2B10	33.71 b	4.00 b	43.49 a	6.60 a	27.94 a	4.17 a
I2B20	36.15 a	4.85 a	41.77 a	6.65 a	27.81 a	4.21 a
I2B30	31.18 b	4.29 ab	28.91 b	4.8 b	27.89 a	4.57 a
I3B0	22.8 b	2.36 c	29.75 c	4.13 d	22.41 c	3.3 c
I3B10	39.31 a	4.58 a	43.39 a	6.49 a	34.51 a	5.26 a
I3B20	36.02 a	4.31 a	35.75 b	5.49 b	32.96 a	5.2 a
I3B30	29.19 b	3.77 b	32.27 bc	5.00 c	26.56 b	4.38 b
I4B0	29.92 b	2.92 b	27.85 c	3.66 b	23.45 c	3.1 b
I4B10	34.68 a	3.52 a	37.16 a	5.44 a	31.07 a	4.27 a
I4B20	34.10 a	3.59 a	34.52 b	5.04 a	30.50 a	4.34 a
I4B30	31.6 b	3.48 a	33.67 b	5.29 a	25.76 b	3.85 ab
ANOVA						
I	*	*	**	*	**	*
B	**	**	*	*	*	**
I × B	*	NS	*	*	**	**

Note: Different letters represent statistical differences between treatments with a significance level of *p* < 0.05 in the LSD test; * indicates that it is significant at the level of 0.05; ** indicates that it is significant at the level of 0.01; I is the irrigation amount; B represents the biochar application rates.

**Table 5:** Stability and sustainability index of sugar beet yield and biomass.

Treatment	Sugar Beet Yield		Sugar Beet Biomass	
	*SYI*	*CV*	*SBI*	*CV*
I1B0	0.68	13.77	0.56	26.15
I1B10	0.82	8.98	0.75	14.32
I1B20	0.78	9.68	0.66	20.75
I1B30	0.73	11.48	0.63	24.11
I2B0	0.66	16.75	0.63	19.76
I2B10	0.83	6.75	0.76	9.99
I2B20	0.79	9.12	0.75	11.99
I2B30	0.76	12.39	0.57	17.98
I3B0	0.64	17.17	0.4	32.02
I3B10	0.89	4.77	0.81	11.44
I3B20	0.85	7.55	0.76	11.88
I3B30	0.77	11.05	0.61	20.64
I4B0	0.74	9.75	0.52	26.41
I4B10	0.84	7.35	0.72	12.21
I4B20	0.82	8.09	0.64	19.72
I4B30	0.78	10.16	0.58	18.6

Note: *SYI* is the sustainable yield index; *SBI* is the sustainable biomass index; *CV* is the coefficient of variance.

## Data Availability

Data will be made available upon request.

## Abbreviations

ET_0_, daily reference crop evapotranspiration; ETc, actual crop evapotranspiration; DAS, days after sowing; LAI, leaf area index; IWUE, irrigation water use efficiency; SYI, sustainable yield index; SBI, sustainable biomass index; CV, coefficient of variance; EB, economic benefit; GR, germination rate.
